# Pharmacological Evaluation of Ticagrelor and Aspirin Versus Clopidogrel and Aspirin Pretreatment on Infarct Artery Flow in Patients with Acute STEMI

**DOI:** 10.3390/ph18121856

**Published:** 2025-12-05

**Authors:** Miljan Opancina, Valentina Opancina, Miloš N. Milosavljević, Ana V. Pejčić, Milos Stepovic, Zoran Jovic

**Affiliations:** 1 Clinic of Cardiology, Military Medical Academy, University of Defense, 11000 Belgrade, Serbia; 2 Faculty of Medical Sciences, University of Kragujevac, 34000 Kragujevac, Serbia; 3 Department of Radiology, Faculty of Medical Sciences, University of Kragujevac, 34000 Kragujevac, Serbia; 4 Department of Pharmacology and Toxicology, Faculty of Medical Sciences, University of Kragujevac, 34000 Kragujevac, Serbia; 5 Department of Anatomy, Faculty of Medical Sciences, University of Kragujevac, 34000 Kragujevac, Serbia

**Keywords:** STEMI, dual antiplatelet therapy, ticagrelor, clopidogrel, TIMI flow, PCI

## Abstract

**Background and Objectives**: Dual antiplatelet therapy (DAPT) with aspirin and a P2Y12 inhibitor is standard in ST-segment elevation myocardial infarction (STEMI). Guidelines favor ticagrelor over clopidogrel, but their effect on infarct artery flow prior to percutaneous coronary intervention (PCI) remains debated. Objective was to compare the effects of aspirin + clopidogrel versus aspirin + ticagrelor pretreatment on infarct artery Thrombolysis in Myocardial Infarction (TIMI) flow in STEMI patients. **Materials and Methods**: This retrospective cohort study included first-time STEMI patients ≥ 18 years admitted to the Military Medical Academy, Belgrade (January 2016–January 2022), who received pretreatment with aspirin + clopidogrel or aspirin + ticagrelor and underwent PCI. TIMI flow was graded before and after PCI. Primary outcomes were pre- and post-PCI TIMI flow; secondary outcome was in-hospital mortality. **Results**: Of 299 STEMI patients, 174 received aspirin + ticagrelor and 125 received aspirin + clopidogrel. Pre-PCI TIMI flow was significantly higher in the ticagrelor group (*p* < 0.001), while post-PCI TIMI flow (*p* = 0.056) and in-hospital mortality (*p* = 0.083) did not significantly differ between groups. After exclusion of patients receiving glycoprotein IIb/IIIa inhibitors, the difference in PCI TIMI flow grade after PCI became statistically significant (*p* = 0.007), favoring the aspirin + ticagrelor group. In multivariate analysis, male gender, drug-eluting stent implantation, and glycoprotein IIb/IIIa inhibitor use were independently associated with reduced in-hospital mortality. **Conclusions**: In STEMI patients, ticagrelor-based DAPT was associated with better initial coronary flow compared to clopidogrel. However, this advantage was not evident after PCI. Male gender, drug-eluting stent implantation, and glycoprotein IIb/IIIa inhibitor use were associated with improved survival.

## 1. Introduction

Clinically, MI is classified into two principal categories: ST-elevation myocardial infarction (STEMI) and non-ST-elevation myocardial infarction (NSTEMI). Unstable angina, often a precursor to MI, is included within the spectrum of acute coronary syndromes (ACS) due to its pathophysiological proximity to infarction events [1].

Globally, STEMI affects over 3 million people annually, with a higher incidence in high-income countries. A meta-analysis reported MI prevalence of 3.8% in individuals under 60 years and 9.5% in those aged 60 and above [2].

Recent epidemiological data indicate a declining trend in STEMI incidence across several European nations and the United States [3].

Patients with acute chest pain and persistent (>20 min) ST-segment elevation reflecting acute total or subtotal coronary occlusion develop AMI with ST-segment elevation [4]. The current standard of care for acute STEMI is immediate reperfusion with primary percutaneous coronary intervention (PCI) or, if not available in time, fibrinolytic therapy. Selected patients (without side-branch occlusion) with STEMI who are at elevated risk for the no-reflow phenomenon may represent a viable population for a novel therapeutic strategy involving deferred stenting [5].

Dual antiplatelet therapy (DAPT) is recommended for patients with STEMI undergoing PCI to prevent ischemic complications such as stent thrombosis and recurrent myocardial infarction [6,7]. By combining aspirin with a P2Y12 inhibitor, DAPT enhances platelet inhibition and reduces thrombotic risk, particularly in the early post-PCI period. However, this benefit is accompanied by an increased risk of bleeding, especially in elderly or comorbid patients, with bleeding recognized as an independent predictor of mortality [8,9]. Early administration of DAPT, consisting of aspirin and a P2Y12 receptor inhibitor, is essential to suppress thrombus progression and is associated with improved survival but also with complications such as bleeding [10].

European guidelines suggest that the earliest possible use of DAPT is preferable to achieve rapid platelet inhibition in STEMI, while discouraging pretreatment with prasugrel, and studies on pretreatment with ticagrelor or clopidogrel have conflicting results [11]. One published study suggests that early DAPT pretreatment in STEMI improves left ventricular function but not coronary flow (TIMI), as there was no difference in TIMI flow before PCI (*p* = 0.653) and after PCI (*p* = 0.205) in the group with early and late DAPT administration (aspirin and clopidogrel) [10].

Large randomized trials have demonstrated that adding clopidogrel to aspirin in patients with acute myocardial infarction significantly improves clinical outcomes by reducing the risk of ischemic complications, including recurrent myocardial infarction and major vascular events, and contributes to lower in-hospital and short-term mortality [12,13]. The optimal choice and timing of P2Y12 inhibitor pretreatment in STEMI remain debated. Ticagrelor provides faster and more potent platelet inhibition compared to clopidogrel, potentially reducing thrombotic risk before PCI. However, clinical trials have shown no consistent improvement in pre-PCI coronary reperfusion or mortality with ticagrelor, highlighting the need to compare its effects directly with clopidogrel in real-world settings [14,15]. Thus, earlier guidelines recommend early ticagrelor use if PCI is planned but emphasize individualized treatment based on patient risk and timing [16]. The most current 2023 ESC Guidelines revised the recommendation on routine pretreatment with P2Y12 inhibitors to the level of consideration.

The aim of this study was to examine the impact of dual antiplatelet therapy (aspirin with clopidogrel, and aspirin with ticagrelor) in pretreatment on infarct artery flow in patients with acute myocardial infarction with ST elevation.

## 2. Results

A total of 299 STEMI patients were included in the study: 125 patients received a combination of aspirin and clopidogrel, while 174 patients received a combination of aspirin and ticagrelor. The study population characteristics are reported in Table 1.

Patients who received aspirin and ticagrelor were significantly younger than patients who received aspirin and clopidogrel. PCI-treated target vessel more frequently included left anterior descending artery (LAD) in patients who received aspirin and ticagrelor, while ramus circumflexis (RCX) was more frequently targeted in patients who received aspirin and clopidogrel. Bare-metal stent was more frequently used in patients treated with aspirin and clopidogrel, while drug-eluting stent was more frequently used in patients treated with aspirin and ticagrelor. Radial access was more frequently employed in patients who received aspirin and clopidogrel, while femoral access was more frequently used in patients who received aspirin and ticagrelor. In our study, 20.1% of STEMI patients received concomitant glycoprotein IIb/IIIa inhibitors, more often in the aspirin + clopidogrel group (24%) than in the aspirin + ticagrelor group (17.2%, *p* = 0.035), reflecting real-world, risk-adapted practice in high thrombotic-risk patients, those with large thrombus burden, or anticipated difficult PCI. This use influenced post-PCI TIMI flow and in-hospital survival, and excluding these patients revealed significant differences in TIMI flow between DAPT groups, suggesting that upfront GPIIb/IIIa use may have masked early perfusion differences. There were no significant differences in gender, comorbidities and other characteristics.

A statistically significant difference (*p* < 0.001) was observed between the groups receiving aspirin and ticagrelor and aspirin and clopidogrel in terms of the initial TIMI flow grade before PCI, and this remained significant after excluding patients who received concomitant glycoprotein IIb/IIIa inhibitors (*p* < 0.001). The group that received aspirin and ticagrelor had a significantly higher TIMI flow grade before PCI compared to the group that received aspirin and clopidogrel, but there was no significant difference between the groups in the TIMI flow grade after PCI (*p* = 0.056). After PCI all patients had either identical or higher TIMI flow grade compared to the grade before PCI. However, after exclusion of patients receiving glycoprotein IIb/IIIa inhibitors, the difference in PCI TIMI flow grade after PCI became statistically significant (*p* = 0.007), favoring the aspirin + ticagrelor group. There was no significant difference (*p* = 0.083) in the frequency of death during hospitalization between the groups of patients that received aspirin and clopidogrel compared to those who received aspirin and ticagrelor (Table 2), and this finding remained non-significant after exclusion of glycoprotein IIb/IIIa inhibitor recipients (*p* = 0.051). Post hoc power analyses for this outcome, calculated using G*Power (version 3.1.9.2), indicated that with the observed effect sizes of w = 0.195 (for all patients) and 0.240 (excluding glycoprotein IIb/IIIa inhibitor recipients), α = 0.05, total sample size of 299 and 239, and 1 degree of freedom, the achieved power was 0.92 and 0.96, respectively, demonstrating that the analyses were sufficiently powered to detect the observed effect.

The results of both univariate and backward stepwise conditional multivariate binary logistic regression from the last step with satisfactory goodness of fit (Hosmer–Lemeshow Chi square = 7.281, df = 6, *p* = 0.296, Cox and Snell R square 0.101, Nagelkerke R square 0.223) evaluating potential predictors of death during hospitalization are shown in Table 3. After adjusting for potential confounders and other independent variables, we identified three protective factors for death during hospitalization: male gender, implantation of a drug-eluting stent, and concomitant use of glycoprotein IIb/IIIa inhibitors. These findings suggest that death during hospitalization is less likely in male STEMI patients who underwent drug-eluting stent implantation and received concomitant glycoprotein IIb/IIIa inhibitors.

The results of both univariate and backward stepwise conditional multivariate binary logistic regression from the last step with satisfactory goodness of fit (Hosmer–Lemeshow Chi square = 1.198, df = 6, *p* = 0.977, Cox and Snell R square 0.115, Nagelkerke R square 0.159) evaluating potential predictors of poor TIMI flow (grade 0 or 1) before PCI are shown in Table 4. After adjusting for potential confounders and other independent variables, hypercholesterolemia emerged as a negative predictor of poor TIMI flow before PCI, while hypertension and use of aspirin and clopidogrel were identified as positive predictors.

The results of both univariate and backward stepwise conditional multivariate binary logistic regression from the last step with satisfactory goodness of fit (Hosmer–Lemeshow Chi square = 0.835, df = 1, *p* = 0.361, Cox and Snell R square 0.095, Nagelkerke R square 0.199) evaluating potential predictors of normal perfusion (TIMI flow grade 3) after PCI are shown in Table 5. After adjusting for potential confounders and other independent variables, we found that use of concomitant glycoprotein IIb/IIIa inhibitors was a negative predictor, while stent implantation was a positive predictor of normal perfusion after PCI.

## 3. Discussion

The results of this retrospective cohort study indicate that the use of aspirin and ticagrelor leads to statistically significantly higher values of initial TIMI flow grade in STEMI patients compared to DAPT pretreatment with aspirin and clopidogrel. After PCI, the observed difference in the effects of different DAPT combinations is lost, as there was no difference in TIMI flow grade between the observed groups of patients with STEMI. Also, there was no difference in the frequency of death during hospitalization between the observed groups of STEMI patients.

The issue of the expediency of DAPT pretreatment in STEMI patients is a subject of frequent cardiological debate. On the one hand, it is believed that this method achieves a faster antiplatelet effect, thereby reducing the risk of ischemic and thrombotic complications that can occur during PCI [17]. On the other hand, early introduction of DAPT in loading doses significantly increases the risk of periprocedural bleeding [17]. Nevertheless, some guidelines for the management of STEMI patients recommend the use of DAPT before PCI due to pharmacological profiles of these drugs [17]. In this spirit, current European and American pretreatment guidelines favor ticagrelor over clopidogrel as part of DAPT with aspirin as a fixed component in the STEMI patient population [17]. The only exceptions are patients with an excessive risk of bleeding or those in whom the STEMI diagnosis is uncertain [18]. However, these recommendations are based on the results of clinical trials, observational studies, and meta-analyses before the new 2023 ESC Guidelines revised recommendations to a, rather, similar level of consideration.

Clopidogrel and ticagrelor essentially belong to the same group of antiplatelet drugs that act by blocking the P2Y12 receptor and thereby preventing the stimulatory effect of ADP on platelet aggregation [19]. However, despite these similarities, there are significant differences in the pharmacodynamic effect between clopidogrel and ticagrelor that have important clinical implications. First of all, ticagrelor has a direct antiplatelet effect, whereas clopidogrel is a prodrug that needs to be activated by liver metabolism on cytochrome P450 2C19 by becoming converted to an active metabolite after its administration [19]. Due to this, ticagrelor has a faster onset of antiplatelet effect compared to clopidogrel, which is of enormous importance in the management of STEMI as an urgent condition [19]. We also indirectly confirmed that ticagrelor has a faster onset of antiplatelet activity compared to clopidogrel, as we showed that STEMI patients who received aspirin and ticagrelor had a significantly higher initial TIMI flow grade compared to the group that received aspirin and clopidogrel. The issue of the speed of onset of antiplatelet action can be of crucial importance in STEMI patients. Therefore, some authors believe that in such situations, patients should be given crushed ticagrelor tablets in order to accelerate the absorption and onset of action of this potent inhibitor [20]. Apart from the delayed onset of action, a considerable percentage of individuals with particular CYP2C19 alleles may have a much reduced antiplatelet effect or complete absence of this effect at all as a result of the initial metabolism of clopidogrel [21]. In addition to its superiority in terms of speed of onset of action, it should be noted that ticagrelor exhibits a stronger antiplatelet effect than clopidogrel [19]. This is confirmed by the results of a meta-analysis conducted by Shabab et al., which showed that ticagrelor as part of DAPT is superior to clopidogrel in terms of reducing overall mortality and the risk of stroke and myocardial infarction in patients with acute coronary syndrome [22]. On the other hand, the more potent antiplatelet effect of ticagrelor is also the reason for the more frequent occurrence of bleeding during the use of this drug compared to clopidogrel [22]. The difference in mortality of STEMI patients between the ticagrelor and clopidogrel groups in our study was not statistically significant (6.9% vs. 13.6%), but its clinical significance cannot be disputed, given that there was an almost twofold reduction in relative risk. It is also important to emphasize that ticagrelor has been shown in various pharmacoeconomic settings to be a cost-effective option compared to clopidogrel in patients with ACS [23,24,25].

Other authors have also confirmed that ticagrelor leads to a significantly higher initial TIMI flow grade compared to clopidogrel as part of DAPT pretreatment preceding PCI in STEMI patients [26,27,28,29]. Our findings show that ticagrelor improves initial TIMI flow compared to clopidogrel, independent of GP IIb/IIIa inhibitor use, while both treatment choice and patient factors influence myocardial perfusion in STEMI patients undergoing PCI. Better initial TIMI flow is a good prognostic parameter in STEMI patients associated with smaller size of infarction and microvascular occlusion [30]. Also, similar to our results, other studies have shown that there was no difference in final TIMI flow grade between groups of STEMI patients receiving ticagrelor and clopidogrel as part of DAPT pretreatment [27,28,29,30]. It is important to note that all STEMI patients in our study, both those receiving ticagrelor and those receiving clopidogrel, had equal or higher TIMI flow grades after PCI compared to the grades before PCI. Our results show that implantation of drug eluting stents plays a crucial role in achieving optimal post-PCI TIMI flow, while concomitant GP IIb/IIIa inhibitor use was associated with lower post-PCI TIMI 3 flow after adjustment for other factors, highlighting the need to consider both interventional strategies and baseline clinical characteristics when assessing myocardial perfusion outcomes in STEMI patients on contemporary DAPT. It is known that higher values of TIMI flow after PCI are associated with a lower risk of stent thrombosis, reinfarction, cardiogenic shock, and 30-day and 180-day mortality [31]. Observational and cardiac magnetic resonance (CMR) studies consistently show that higher pre-PCI TIMI flow predicts smaller infarct size and less microvascular obstruction (MVO) [30]. MVO and infarct size themselves are powerful prognostic markers for mortality and heart-failure hospitalization after STEMI [32,33].

However, pharmacologic strategies that improve early epicardial patency have not reliably yielded downstream reductions in infarct size/MVO or hard outcomes. Small imaging trials and pooled analyses suggest ticagrelor may enhance microcirculatory surrogates (better corrected TIMI frame count or myocardial blush grade) and, in some CMR studies, smaller infarcts and less MVO compared with clopidogrel potentially via adenosine-mediated effects, but results are heterogeneous and not accompanied by clear mortality benefit [34,35]. Moreover, when platelet inhibition was intensified at reperfusion with IV cangrelor on top of oral ticagrelor, CMR-measured infarct size and MVO were unchanged, illustrating the difficulty of converting improved pharmacodynamics into myocardial salvage in contemporary PCI pathways [36].

Taken together, our findings align with the broader literature, and better pre-PCI TIMI flow correlates with smaller myocardial injury [30], but demonstrating that ticagrelor-driven improvements in initial flow translate into less MVO, smaller infarcts, or superior long-term outcomes has proved challenging [15,34,35,36]. Future work should incorporate standardized CMR endpoints (infarct size, MVO) and longer follow-up to clarify whether early flow improvements with ticagrelor yield durable clinical benefit in modern STEMI care.

However, whenever the antiaggregatory effect of ticagrelor is analyzed in the STEMI patient population, the results of the ATLANTIC study should be mentioned, which showed that early administration of ticagrelor before PCI does not lead to a significant improvement in reperfusion of the culprit artery [15].

The results of our regression analysis identified protective factors that contribute to the survival of STEMI patients undergoing PCI, which have also been identified by other researchers. Thus, we have shown that the use of antiplatelet drugs from the group of IIb/IIIa inhibitors during PCI reduces the rate of in-hospital mortality in STEMI patients. Glycoprotein IIb/IIIa inhibitors are potent antiplatelet drugs whose single periprocedural administration reduces the risk of 30-day and 180-day mortality and recurrent ischemic events in STEMI patients [37]. Another identified factor that reduces in-hospital mortality of STEMI patients undergoing PCI in our study was the use of drug-eluting stents. Wang et al. showed that the use of drug-eluting stents contributes to a statistically significant greater reduction in the risk of revascularization and reinfarction compared to the use of bare-metal stents in STEMI patients [38]. Finally, we have shown that male gender contributes to the survival of STEMI patients, which has also been previously confirmed. Thus, Marinsek et al. showed that the frequency of in-hospital mortality after PCI was significantly higher in female STEMI patients compared to males (14% vs. 8%) [39]. There are several clinical reasons why in-hospital mortality is higher in female STEMI patients, and one of them could be in-hospital bleeding. Namely, Spadafora et al., in a large study that included 1060 patients with ACS who experienced in-hospital bleeding, showed that female gender is a significant risk factor for in-hospital bleeding [40].

Patients receiving ticagrelor in our cohort were, on average, younger and more frequently treated with newer generation drug eluting stents, both of which independently influence outcomes after STEMI [41,42]. Although we adjusted for these confounders in multivariable models, residual confounding cannot be excluded. This may partly explain why ticagrelor was associated with improved pre-PCI TIMI flow without a corresponding reduction in mortality or hard clinical endpoints. The landmark PLATO trial demonstrated a significant mortality benefit of ticagrelor over clopidogrel in a broad ACS population [43]. Our findings support the current guideline preference for ticagrelor as a potent oral P2Y12 inhibitor, but they also highlight that in contemporary STEMI care, where baseline patient factors and procedural characteristics such as stent type strongly influence outcomes.

Our study evaluated the impact of administering dual antiplatelet therapy (DAPT), including a P2Y12 inhibitor, within 2 h prior to primary PCI in STEMI patients. Notably, our enrollment concluded before the publication of the 2023 ESC Guidelines for the Management of Acute Coronary Syndromes, which revised the recommendation on routine pretreatment with P2Y12 inhibitors in STEMI. Specifically, the guidelines downgraded this strategy to a Class IIb (may be considered), Level of Evidence B, reflecting uncertainty around clinical benefit and potential bleeding risk when pretreatment is given without knowledge of coronary anatomy [44]. In contrast to trials like ATLANTIC [15], which evaluated shorter pretreatment intervals (approximately 30 min), our 2 h window allowed more time for drug absorption and antiplatelet activity, particularly with faster-acting agents such as ticagrelor or prasugrel. Our findings suggest that earlier administration of DAPT, when timed appropriately, may still offer clinical benefit, especially in settings with longer prehospital transfer times. However, in light of current guideline shifts, our results highlight the need to tailor pretreatment decisions based on patient risk profile, drug choice, and anticipated PCI timing, rather than apply a routine pretreatment strategy to all STEMI patients.

Although our results are coherent with those of other researchers, it should be noted that our study has certain limitations. The retrospective design and relatively small sample size pose two of the most important limitations to the generalizability of our research results. Additional limitation of this study is the absence of time-to-event data for death during hospitalization, which prevented the use of Kaplan–Meier survival analysis to evaluate survival times. Due to the retrospective study design and variability in documentation, the exact timing from DAPT administration to PCI was not consistently available for all patients. Furthermore, the lack of long-term outcome data limits our ability to assess the sustained impact of the findings beyond the hospitalization period. Potential unmeasured confounders may also have influenced the results despite adjustment for known factors. Importantly, our dataset did not capture the interval between symptom onset and PCI or details of prehospital management, such as the use of crushed ticagrelor or opioid medications. Genetic polymorphisms affecting clopidogrel metabolism, particularly CYP2C19 variants, were also not assessed. These factors could represent additional confounders influencing treatment response and outcomes. Additionally, bleeding complications could not be analyzed, as they were not systematically documented. The relatively small sample size and limited number of outcome events restricted the use of advanced methods like propensity score matching to further control for baseline differences. Since the study was conducted at a single tertiary care center in Serbia, caution should be exercised when generalizing these findings to other populations or healthcare settings. Finally, a significant proportion of patients in our cohort received bare-metal stents (BMS), which represents real-world practice during the study period rather than deviation from guideline recommendations. At the time, BMS were occasionally selected in patients with high bleeding risk or those requiring a shorter duration of dual antiplatelet therapy, as well as in cases influenced by logistic or reimbursement constraints. Our findings showing better outcomes with drug-eluting stents (DES) align with current ESC guidelines and reinforce their prognostic advantage. Nonetheless, inclusion of BMS-treated patients provides valuable insight into outcomes in higher-risk subgroups and historical practice patterns.

## 4. Materials and Methods

Our study was designed as a clinical observational, retrospective cohort study. It was approved by the Ethics Committee of the Military Medical Academy in Belgrade.

The study included all patients who were firstly diagnosed with acute myocardial infarction with ST elevation and who received DAPT (aspirin + clopidogrel or aspirin + ticagrelor) as pretreatment, after which they were treated with PCI and hospitalized at the Military Medical Academy in the period from January 2016 to January 2022.

Patients were divided into two cohorts depending on the type of DAPT received in pretreatment:

1. Patients who received aspirin and clopidogrel therapy in pretreatment;
2. Patients who received aspirin and ticagrelor therapy in pretreatment.

Inclusion criteria were: ≥18-year-old patients, diagnosed with STEMI for the first time; received pre-treatment with DAPT (aspirin + clopidogrel or aspirin + ticagrelor); admitted at the Military Medical Academy in Belgrade during observed period, treated with PCI; measured TIMI flow before and after PCI.

The diagnosis of AIM requires a combination of criteria, i.e., detection of an increase and/or decrease in a cardiac biomarker (preferably high-sensitivity cardiac troponin (hs-cTn) T or I, with at least one value above the 99th percentile of the upper reference limit) and at least one of the following criteria: symptoms of myocardial ischemia; new ischemic ECG changes; early convex ST segment elevation in 2 adjacent leads > 1 mm, most often with ST depression in opposite leads; imaging evidence of loss of viable myocardium or new regional wall motion abnormalities in a pattern consistent with ischemic etiology; intracoronary thrombus detected by angiography (1). Patients with acute chest pain and persistent (>20 min) ST-segment elevation reflecting acute total or subtotal coronary occlusion were diagnosed with STEMI, defined according to the 4th Universal Definition of Myocardial Infarction as new ST-segment elevation at the J-point in two contiguous leads with the following thresholds: ≥1.0 mm in all leads other than V2–V3; ≥2.0 mm in men ≥ 40 years, ≥2.5 mm in men < 40 years, and ≥1.5 mm in women [1]. Study exclusion criteria were: acute coronary syndrome without diagnosed STEMI; patient who did not receive aspirin + clopidogrel or aspirin + ticagrelor therapy in pretreatment; patient who did not undergo PCI; pregnancy; participation in another interventional study (participation in an observational study is not an exclusion criterion); comorbidity or severe complications that would prevent adequate treatment of the patient (e.g., cardiogenic shock, cardiopulmonary insufficiency, etc.); incomplete medical documentation.

PCI was performed by experienced medical team of four different interventional cardiologists, with more than 10 years of experience.

During the study period (2016–2022), both drug-eluting stents (DES) and bare-metal stents (BMS) were used, reflecting real-world clinical practice at our center. The choice of stent type was guided by individual patient characteristics, including bleeding risk, anticipated duration of dual antiplatelet therapy, comorbidities, and, in earlier years, reimbursement and logistic factors.

The TIMI scale was used in the evaluation of patients. TIMI flow was evaluated during coronary angiography and immediately after PCI by the same physician performing the intervention. The TIMI scale was used to evaluate coronary flow through the infarcted artery during coronary angiography and immediately after PCI, assessed by the same interventional cardiologist performing the procedure. TIMI flow was graded from 0 to 3 according to standard definitions [45]:

1. TIMI 0—no perfusion: no antegrade flow beyond the site of occlusion.
2. TIMI 1—penetration without perfusion: contrast passes beyond the occlusion but fails to opacify the distal coronary bed.
3. TIMI 2—partial perfusion: contrast opacifies the distal vessel but flow is slower than normal.
4. TIMI 3—complete perfusion: normal flow, comparable to non-infarcted coronary arteries.

Dual antiplatelet therapy is given immediately after the diagnosis of STEMI.

The study outcomes were: difference in coronary flow through the infarcted artery (TIMI) before and after PCI and death outcome during hospitalization.

Coronary flow through the infarcted artery was measured using the TIMI scale, with values from 0 to 3. TIMI—the degree of coronary flow was established to provide a uniform and consistent method of recording epicardial perfusion on coronary arteriography [45].

Other variables examined in the study were: type of DAPT: aspirin 300 mg and clopidogrel 600 mg versus aspirin 300 mg and ticagrelor 180 mg; sociodemographic factors; concomitant diseases; interventional procedure used to treat the infarcted artery (primary PCI); coronary angiography findings; previous intervention—coronary bypass; characteristics of PCI; vascular access to the intervention; concomitant therapy. No patients in our study received thrombolytic therapy.

Due to the retrospective study design and variability in documentation, the exact timing from DAPT administration to PCI was not consistently available for all patients. However, based on the local guideline recommendations, DAPT was administered within 2 h.

Statistical Program for Social Sciences (SPSS version 18) was used to conduct all statistical analyses. The data were initially analyzed by descriptive statistics. Normally distributed continuous variables were expressed as mean ± standard deviation (SD), while non-normally distributed continuous variables were presented as median and interquartile range (Q1–Q3). Categorical variables were presented as frequencies and percentages (%). Kolmogorov–Smirnov test was used to test the normality of the continuous variables. The differences in continuous variables between the groups were tested by independent group *t*-test (for normally distributed variables) or Mann–Whitney U test (for non-normally distributed variables). To assess differences in categorical variables we used Chi-squared (χ^2^) test or Fisher’s exact test, depending on assumptions of which tests were satisfied. A post hoc power analysis for the chi-squared test was conducted using G*Power (version 3.1.9.2) to assess the achieved statistical power for the outcome of death during hospitalization, based on the observed effect size, sample size, and significance level. To assess the influence of potential predictors on the occurrence of poor TIMI flow (grade 0 or 1) before PCI, normal perfusion (TIMI flow grade 3) after PCI, and death during hospitalization we calculated crude and adjusted odds ratios (ORs) with corresponding 95% confidence intervals (CIs) using univariate and backward stepwise conditional multivariate binary logistic regression analysis. Variables for inclusion in the multivariate logistic regression models were selected based on a combination of statistical significance and clinical relevance. Specifically, variables that showed a *p*-value < 0.05 in univariate analysis were considered for entry, along with additional clinically important covariates that could plausibly act as confounders, even if not statistically significant in univariate testing. This approach ensured adjustment for both data-driven and clinically justified predictors. Model fit and assumptions were evaluated using standard diagnostic methods. To assess multicollinearity among independent variables included in the logistic regression models, we calculated variance inflation factors (VIFs) using a linear regression model with the same set of predictors. Although VIFs are not directly available in logistic regression in SPSS, this approach provides a valid diagnostic of multicollinearity. All VIF values across all steps in reported models were below 2, indicating no evidence of multicollinearity among the predictors. A *p*-value less than 0.05 was considered statistically significant.

## 5. Conclusions

In conclusion, the use of ticagrelor in combination with aspirin before PCI was accompanied by significantly higher initial TIMI flow grades compared to the use of clopidogrel and aspirin in STEMI patients. On the other hand, there seems to be no significant difference regarding the final TIMI flow grades after PCI between the two estimated types of DAPT pretreatment in STEMI patients. The administration of glycoprotein IIb/IIIa inhibitors during PCI, male gender, and the application of drug-eluting stents are factors that reduce the risk of mortality during hospitalization of STEMI patients.

## Figures and Tables

**Table 1:** Characteristics of the study population.

Variable	All Patients (*n* = 299)	Aspirin + Clopidogrel (*n* = 125)	Aspirin + Ticagrelor (*n* = 174)	*p*-Value
Age (years)	64.1 ± 12.4	66.1 ± 12.4	62.6 ± 12.2	0.015 ^1^*
Gender				
Male	207 (69.2%)	85 (68.0%)	122 (70.1%)	0.792 ^3^
Female	92 (30.8%)	40 (32.0%)	52 (29.9%)	
Diabetes	88 (29.4%)	40 (32.0%)	48 (27.6%)	0.611 ^3^
Hypercholesterolemia	202 (67.6%)	86 (68.8%)	116 (66.7%)	0.881 ^3^
Hypertension	241 (80.6%)	101 (80.8%)	140 (80.5%)	0.758 ^3^
Active smoker	166 (55.5%)	62 (49.6%)	104 (59.8%)	0.097 ^3^
Multivessel disease	171 (57.2%)	73 (58.4%)	98 (56.3%)	1.000 ^3^
Number of PCI-treated target vessels				
One target vessel	245 (81.9%)	102 (81.6%)	143 (82.2%)	0.913 ^4^
Two target vessels	34 (11.4%)	13 (10.4%)	21 (12.1%)	
Three target vessels	1 (0.3%)	0 (0.0%)	1 (0.6%)	
PCI-treated target vessel				
RCA	114 (38.1%)	51 (40.8%)	63 (36.2%)	0.363 ^3^
LAD	107 (35.8%)	30 (24.0%)	77 (44.2%)	0.001 ^3^*
RCX	42 (14.0%)	25 (20.0%)	17 (9.7%)	0.014 ^3^*
OM	23 (7.7%)	14 (11.2%)	9 (5.2%)	0.073 ^3^
LMCA	5 (1.7%)	1 (0.8%)	4 (2.3%)	0.652 ^4^
Venous graft	5 (1.7%)	1 (0.8%)	4 (2.3%)	0.652 ^4^
Minor vessels (PDA, PLV, D)	16 (5.4%)	6 (4.8%)	10 (5.8%)	0.970 ^3^
Stent implantation	257 (86.0%)	105 (84.0%)	152 (87.4%)	0.512 ^3^
Number of stents	1 (1–2)	1 (1–2)	1 (1–2)	0.467 ^2^
0	42 (14.0%)	20 (16.0%)	22 (12.6%)	0.611 ^4^
1	161 (53.8%)	66 (52.8%)	95 (54.6%)	
2	76 (25.4%)	33 (26.4%)	43 (24.7%)	
3	16 (5.4%)	4 (3.2%)	12 (6.9%)	
4	4 (1.3%)	2 (1.6%)	2 (1.1%)	
Type of stent				
Bare-metal stent	86 (28.8%)	52 (41.6%)	34 (19.5%)	<0.001 ^3^*
Drug-eluting stent	173 (57.9%)	52 (41.6%)	121 (69.5%)	<0.001 ^3^*
Absorb bioresorbable scaffold	4 (1.3%)	2 (1.6%)	2 (1.1%)	1.000 ^4^
Access				
Radial	146 (48.8%)	72 (57.6%)	60 (34.5%)	<0.001 ^4^*
Femoral	132 (44.1%)	48 (38.4%)	98 (56.3%)	
Radial-femoral	18 (6.0%)	4 (3.2%)	14 (8.0%)	
Ulnar	3 (1.0%)	1 (0.8%)	2 (1.1%)	
Concomitant anticoagulant	293 (98.0%)	122 (97.6%)	171 (98.3%)	0.697 ^3^
Heparin	288 (96.3%)	120 (96.0%)	168 (96.6%)	1.000 ^3^
Enoxaparin	5 (1.7%)	2 (1.6%)	3 (1.7%)	1.000 ^4^
Concomitant glycoprotein IIb/IIIa inhibitor	60 (20.1%)	30 (24.0%)	30 (17.2%)	0.035 ^3^*

Abbreviations: D—diagonal artery; LAD—Left anterior descending artery; LMCA—Left main coronary artery; OM—Obtuse marginal artery; PCI—percutaneous coronary intervention; PDA—Posterior descending artery; PLV—Posterior left ventricular artery; RCA—Right coronary artery; RCX—Ramus circumflexis. Results are presented as mean ± standard deviation; median (range) or number (%). ^1^—*t*-test, ^2^—Mann–Whitney U test, ^3^—χ^2^ test, ^4^—Fisher’s Exact Test, *—statistically significant (<0.05).

**Table 2:** Frequency of TIMI flow grade before and after PCI and occurrence of death during hospitalization.

Variable	Including Patients Who Received Concomitant Glycoprotein IIb/IIIa Inhibitor				Excluding Patients Who Received Concomitant Glycoprotein IIb/IIIa Inhibitor			
	All Patients (*n* = 299)	Aspirin + Clopidogrel (*n* = 125)	Aspirin + Ticagrelor (*n* = 174)	*p*-Value	All Patients (*n* = 239)	Aspirin + Clopidogrel (*n* = 95)	Aspirin + Ticagrelor (*n* = 144)	*p*-Value
TIMI flow grade before PCI								
Grade 0 or 1	194 (64.9%)	97 (77.6%)	97 (55.7%)	*p* < 0.001 ^1^*	141 (59.0%)	70 (73.7%)	71 (49.3%)	*p* < 0.001 ^1^*
Grade 2	65 (21.7%)	14 (11.2%)	51 (29.3%)		63 (26.4%)	13 (13.7%)	50 (34.7%)	
Grade 3	40 (13.4%)	14 (11.2%)	26 (14.9%)		35 (14.6%)	12 (12.6%)	23 (16.0%)	
TIMI flow grade after PCI								
Grade 0 or 1	11 (3.7%)	5 (4.0%)	6 (3.4%)	*p* = 0.056 ^1^	5 (2.1%)	3 (3.2%)	2 (1.4%)	*p* = 0.007 ^2^*
Grade 2	23 (7.7%)	15 (12.0%)	8 (4.6%)		13 (5.4%)	10 (10.5%)	3 (2.1%)	
Grade 3	265 (88.6%)	105 (84.0%)	160 (92.0%)		221 (92.5%)	82 (86.3%)	139 (96.5%)	
Death during hospitalization	29 (9.7%)	17 (13.6%)	12 (6.9%)	*p* = 0.083 ^1^	23 (9.6%)	14 (14.7%)	9 (6.2%)	*p* = 0.051 ^1^

Abbreviations: TIMI—thrombolysis in myocardial infarction; PCI—percutaneous coronary intervention; ^1^—χ^2^ test; ^2^—Fisher’s Exact Test; *—statistically significant (<0.05).

**Table 3:** The results of univariate and the last step of backward stepwise conditional multivariate binary logistic regression analysis evaluating potential predictors of death during hospitalization.

Variable	Survived (*n* = 270)	Died (*n* = 29)	Crude OR with 95% CI, *p*-Value	Adjusted ^#^ OR with 95% CI, *p*-Value	VIF
Age (years)	63.4 ± 12.1	69.8 ± 13.4	1.045 (1.011; 1.081), 0.010 *	–	–
Male gender	193 (71.5%)	14 (48.3%)	0.372 (0.172; 0.808); 0.012 *	0.325 (0.119; 0.891), 0.029 *	1.006
Diabetes	78 (28.9%)	10 (34.5%)	1.365 (0.598; 3.115). 0.460	–	–
Hypercholesterolemia	181 (67.0%)	21 (72.4%)	1.450 (0.563; 3.736). 0.441	–	–
Hypertension	217 (80.4%)	24 (82.8%)	1.585 (0.457; 5.500), 0.468	–	–
Active smoker	155 (57.4%)	11 (37.9%)	0.457 (0.204; 1.024), 0.057	–	–
Multivessel disease	154 (57.0%)	17 (58.6%)	2.015 (0.655; 6.201), 0.222	–	–
Two or three PCI-treated target vessels	34 (12.6%)	1 (3.4%)	0.248 (0.033; 1.886), 0.178	–	–
LAD as culprit (PCI-treated target) vessel	30 (24.0%)	77 (44.2%)	1.124 (0.501; 2.524), 0.776	–	–
RCX as culprit (PCI-treated target) vessel	25 (20.0%)	17 (9.7%)	1.327 (0.473; 3.723), 0.591	–	–
Stent implantation	236 (87.4%)	21 (72.4%)	0.378 (0.155; 0.921), 0.032 *	–	–
Number of stents	1 (1–2)	1 (0–1.5)	0.805 (0.491; 1.322), 0.392	–	–
Bare-metal stent	74 (27.4%)	12 (41.4%)	1.870 (0.852; 4.103), 0.119	–	–
Drug-eluting stent	165 (61.1%)	8 (27.6%)	0.242 (0.104; 0.567), 0.001 *	0.192 (0.061; 0.606), 0.005 *	1.352
Absorb bioresorbable scaffold	3 (1.1%)	1 (3.4%)	3.179 (0.320; 31.950), 0.324	–	–
Radial access	141 (52.2%)	5 (17.2%)	0.191 (0.071; 0.514), 0.001 *	0.333 (0.099; 1.122), 0.076	1.510
Femoral access	48 (38.4%)	98 (56.3%)	3.760 (1.608; 8.794), 0.002 *	–	–
Aspirin and clopidogrel	108 (40.0%)	17 (58.6%)	2.125 (0.976; 4.627), 0.058	–	–
Concomitant anticoagulant	264 (97.8%)	29 (100.0%)	–	–	–
Concomitant glycoprotein IIb/IIIa inhibitor	54 (20.0%)	6 (20.7%)	1.092 (0.410; 2.908), 0.861	0.225 (0.062; 0.817), 0.023 *	1.246
TIMI Grade 0 or 1 before PCI	173 (64.1%)	21 (72.4%)	1.472 (0.628; 3.448), 0.374	–	–
TIMI Grade 3 after PCI	242 (89.6%)	23 (79.3%)	0.444 (0.166; 1.182), 0.104	–	–

^#^ Adjusted for: age, male gender, diabetes, hypercholesterolemia, hypertension, active smoker, LAD as culprit (PCI-treated target) vessel, RCX as culprit (PCI-treated target) vessel, stent implantation, number of stents, drug-eluting stent, radial access, aspirin and clopidogrel, concomitant glycoprotein IIb/IIIa inhibitor, TIMI Grade 0 or 1 before PCI, TIMI Grade 3 after PCI. Abbreviations: CI—confidence interval; LAD—Left anterior descending artery; RCX—Ramus circumflexis; TIMI—thrombolysis in myocardial infarction; OR—odds ratio; PCI—percutaneous coronary intervention; VIF—variance inflation factor; *—statistically significant (<0.05).

**Table 4:** The results of univariate and the last step of backward stepwise conditional multivariate binary logistic regression analysis evaluating potential predictors of poor TIMI flow (grade 0 or 1) before PCI.

Variable	Poor TIMI Flow (Grade 0 or 1) Before PCI (*n* = 194)	Good TIMI Flow (Grade 2 or 3) Before PCI (*n* = 105)	Crude OR with 95% CI, *p*-Value	Adjusted ^#^ OR with 95% CI, *p*-Value	VIF
Age (years)	64.6 ± 12.6	63.0 ± 11.9	1.011 (0.991; 1.030), 0.278	–	–
Male gender	133 (68.6%)	74 (70.5%)	0.913 (0.544; 1.532); 0.731	–	–
Diabetes	55 (28.3%)	33 (31.4%)	0.872 (0.517; 1.469). 0.606	–	–
Hypercholesterolemia	120 (61.9%)	82 (78.1%)	0.448 (0.250; 0.806). 0.007 *	0.310 (0.146; 0.657), 0.002 *	1.118
Hypertension	159 (82.0%)	82 (78.1%)	1.364 (0.716; 2.600), 0.345	3.147 (1.381; 7.170), 0.006 *	1.110
Active smoker	104 (53.6%)	62 (59.1%)	0.849 (0.518; 1.391), 0.517	–	–
Multivessel disease	110 (56.7%)	61 (58.1%)	0.633 (0.348; 1.150), 0.133	0.534 (0.271; 1.052), 0.070	1.028
LAD as culprit (PCI-treated target) vessel	66 (34.0%)	41 (39.0%)	0.833 (0.505; 1.375), 0.475	–	–
RCX as culprit (PCI-treated target) vessel	29 (14.9%)	13 (12.4%)	1.285 (0.635; 2.602), 0.486	–	–
Aspirin and clopidogrel	97 (50.0%)	28 (26.7%)	2.750 (1.641; 4.607), <0.001 *	2.785 (1.486; 5.219), 0.001 *	1.001
Concomitant anticoagulant	188 (96.9%)	105 (100.0%)	–	–	–

^#^ Adjusted for: age, male gender, diabetes, hypercholesterolemia, hypertension, active smoker, multivessel disease, LAD as culprit (PCI-treated target) vessel, RCX as culprit (PCI-treated target) vessel, aspirin and clopidogrel, concomitant anticoagulant. Abbreviations: CI—confidence interval; TIMI—thrombolysis in myocardial infarction; OR—odds ratio; PCI—percutaneous coronary intervention; VIF—variance inflation factor; *—statistically significant (<0.05).

**Table 5:** The results of univariate and the last step of backward stepwise conditional multivariate binary logistic regression analysis evaluating potential predictors of normal perfusion (TIMI flow grade 3) after PCI.

Variable	Non-Normal Perfusion (TIMI Flow Grade 0–2) After PCI (*n* = 34)	Normal Perfusion (TIMI Flow Grade 3) After PCI (*n* = 265)	Crude OR with 95% CI, *p*-Value	Adjusted ^#^ OR with 95% CI, *p*-Value	VIF
Age (years)	68.2 ± 13.6	63.5 ± 12.2	0.969 (0.940; 0.999), 0.042 *	–	–
Male gender	20 (58.8%)	187 (70.6%)	1.678 (0.807; 3.490); 0.166	–	–
Diabetes	10 (29.4%)	78 (29.4%)	0.876 (0.392; 1.959). 0.748	–	–
Hypercholesterolemia	24 (70.6%)	178 (67.2%)	0.593 (0.233; 1.510). 0.274	–	–
Hypertension	27 (79.4%)	214 (80.7%)	0.967 (0.352; 2.657), 0.947	–	–
Active smoker	14 (41.2%)	152 (57.4%)	1.687 (0.789; 3.605), 0.177	–	–
Multivessel disease	17 (50.0%)	154 (58.1%)	1.104 (0.438; 2.782), 0.834	–	–
Two or three PCI-treated target vessels	2 (5.9%)	33 (12.4%)	1.959 (0.444; 8.640), 0.375	–	–
LAD as culprit (PCI-treated target) vessel	7 (20.6%)	100 (37.7%)	1.974 (0.809; 4.815), 0.135	–	–
RCX as culprit (PCI-treated target) vessel	5 (14.7%)	37 (14.0%)	0.792 (0.283; 2.213), 0.656	–	–
Stent implantation	20 (58.8%)	237 (89.4%)	5.925 (2.696; 13.020), <0.001 *	6.825 (2.166; 21.508), 0.001 *	1.017
Number of stents	1 (0–1)	1 (1–2)	2.329 (1.360; 3.989), 0.002 *	–	–
Bare-metal stent	8 (23.5%)	78 (29.4%)	1.356 (0.588; 3.125), 0.475	–	–
Drug-eluting stent	12 (35.3%)	161 (60.8%)	2.838 (1.347; 5.980), 0.006 *	–	–
Absorb bioresorbable scaffold	0 (0.0%)	4 (1.5%)	–	–	–
Radial access	12 (35.3%)	134 (50.6%)	1.875 (0.892; 3.944), 0.097	–	–
Femoral access	19 (55.9%)	113 (42.6%)	0.587 (0.286; 1.205), 0.147	–	–
Aspirin and ticagrelor	14 (41.2%)	160 (60.4%)	2.177 (1.053; 4.499), 0.036 *	–	–
Concomitant anticoagulant	33 (97.1%)	260 (98.1%)	1.576 (0.179; 13.903), 0.682	–	–
Concomitant glycoprotein IIb/IIIa inhibitor	16 (47.1%)	44 (16.6%)	0.209 (0.094; 0.467), <0.001 *	0.218 (0.084; 0.563), 0.002 *	1.017

^#^ Adjusted for: age, male gender, diabetes, hypercholesterolemia, hypertension, active smoker, LAD as culprit (PCI-treated target) vessel, RCX as culprit (PCI-treated target) vessel, stent implantation, number of stents, drug-eluting stent, radial access, aspirin and ticagrelor, concomitant glycoprotein IIb/IIIa inhibitor. Abbreviations: CI—confidence interval; LAD—Left anterior descending artery; RCX—Ramus circumflexis; TIMI—thrombolysis in myocardial infarction; OR—odds ratio; PCI—percutaneous coronary intervention; VIF—variance inflation factor; *—statistically significant (<0.05).

## Data Availability

The original contributions presented in this study are included in the article. Further inquiries can be directed to the corresponding author.
